# Identification of antibiotic resistance genes in fecal microbiota selected donors during the establishment of a biobank in the south of Brazil

**DOI:** 10.3389/frabi.2026.1905540

**Published:** 2026-09-25

**Authors:** Lucas de Figueiredo Soveral, Liandra Raphaella de Lima Holanda, Izadora Borgmann Frizzo Assunção, Leticia Gonçalves Gomes, Isabella Bittencourt de Souza, Gabriella de Souza Rodrigues, Patrícia Almeida Vanny, Oscar Bruna-Romero, Jussara Kasuko Palmeiro, Mara Cristina Scheffer, Thais Cristine Marques Sincero, Carlos Rodrigo Zárate-Bladés

**Affiliations:** 1 Laboratory of Immunoregulation, Department of Microbiology, Immunology, and Parasitology, Federal University of Santa Catarina, Florianópolis, Brazil; 2 Center for Dysbiosis Control, Federal University of Santa Catarina, Florianópolis, Brazil; 3 National Institute of Science and Technology for Mucosa and Skin, Belo Horizonte, Brazil; 4 Laboratory of Molecular Microbiology, Department of Clinical Analysis, Federal University of Santa Catarina, Florianópolis, Brazil; 5 Clinical Specialties Unit, University Hospital Polydoro Ernani de São Thiago (HU-UFSC/EBSERH), Federal University of Santa Catarina, Florianópolis, Brazil; 6 Laboratory of Applied Immunology, Department of Microbiology, Immunology, and Parasitology, Federal University of Santa Catarina, Florianópolis, Brazil; 7 Laboratory of Molecular Biology, Microbiology and Serology, University Hospital Polydoro Ernani de São Thiago, Federal University of Santa Catarina, Florianópolis, Brazil

**Keywords:** antimicrobial resistance, biosafety, fecal microbiota transplantation, metagenomics, microbiota-based therapies, resistome

## Abstract

Fecal microbiota transplantation (FMT) is an effective therapy for recurrent *Clostridioides difficile* infection (rCDI) and is increasingly explored for other dysbiosis-related disorders. However, its implementation as a regulated therapeutic strategy requires robust donor screening, biosafety frameworks, and standardized processing workflows. Here, we describe the establishment of the first fecal microbiota biobank in southern Brazil and evaluate the incorporation of metagenomic sequencing as a complementary layer of donor characterization and safety assessment. A structured donor selection pipeline based on international guidelines was implemented, integrating clinical screening, biochemical and serological testing, and microbiological analyses. Of 100 screened candidates, only four donors met all eligibility criteria and were included in the biobank, highlighting the stringency of the selection process. Shotgun metagenomic sequencing revealed a diverse resistome across all donors, including a shared core set of resistance genes alongside marked interindividual variability. Dominant resistance determinants included tetracycline-associated genes, *ermF*, CfxA-type β-lactamases, and aminoglycoside-modifying enzymes, which were associated with specific gut taxa. Genomic context analysis further identified four ARG-containing contigs with features compatible with potential mobility, although active horizontal gene transfer was beyond the scope of this study. Notably, *tet(Q)* and *ermF* were predominantly associated with *Bacteroides fragilis*, supporting its role as a reservoir of resistance determinants in the gut microbiota. These findings support the use of metagenomic profiling as a complementary approach to donor characterization and risk assessment rather than as a standalone criterion for donor exclusion. Overall, this study provides a framework for microbiota biobank implementation and highlights the potential of metagenomic analysis to complement conventional biosafety screening in FMT.

## 1 Introduction

Fecal microbiota transplantation (FMT) has consolidated itself as one of the most effective therapeutic interventions for rCDI, with success rates exceeding 90% (van Nood et al., 2013; Quraishi et al., 2017). Beyond this indication, FMT has progressively gained attention as a potential therapeutic strategy for a wide spectrum of disorders linked to intestinal dysbiosis, including inflammatory, metabolic, and immune-mediated diseases (Smits et al., 2013; Qu et al., 2022; Soveral et al., 2022).

Despite its clinical relevance, FMT poses singular challenges from a technical and safety perspective. Unlike conventional pharmaceuticals or biologics composed of defined molecular entities, FMT involves the transfer of a complex and dynamic biological ecosystem, whose composition and functional attributes remain only partially characterized (Khoruts and Sadowsky, 2016; Vaughn et al., 2016; Xiao et al., 2020). This intrinsic complexity complicates standardization, traceability and quality control, fundamental requirements for the development of safe and scalable therapeutic products (Cammarota et al., 2017; Chen et al., 2021).

In this context, fecal microbiota biorepositories and stool banks have emerged as essential infrastructures for the consolidation of FMT as a regulated therapy. These platforms aim to centralize donor recruitment, ensure standardized screening, enable controlled processing and storage, and support the traceability of material used for clinical application (Terveer et al., 2017; Kragsnaes et al., 2020). However, the implementation of such systems remains heterogeneous across countries, reflecting differences in regulatory environments, technological capacity, and clinical practice (Cammarota et al., 2017; Haifer et al., 2020; Terra et al., 2020).

Current donor screening protocols are primarily designed to exclude known pathogens and high-risk clinical conditions (Woodworth et al., 2017). While indispensable, these strategies are limited to predefined targets and do not fully encompass the functional and genetic complexity of donor microbiota (Bibbò et al., 2020; Liu et al., 2026). As FMT expands toward new clinical indications and increasingly vulnerable patient populations, the need for more comprehensive a approaches to biosafety becomes increasingly evident (Ianiro et al., 2022; Liu et al., 2025).

Within this, the present study describes the establishment of the first fecal microbiota biobank in Santa Catarina and explores the use of metagenomic sequencing as a complementary tool for safety assessment, not as a definitive safety criterion, but as a way for expanding quality control strategies in microbiota-based therapies.

## 2 Materials and methods

### 2.1 Study design

This study was designed as a descriptive and methodological investigation aimed at establishing the first fecal microbiota biobank in the state of Santa Catarina, Brazil, intended to support the future clinical use of fecal microbiota transplantation (FMT) in selected diseases. The study was structured in three main sequential phases: (i) development of donor screening tools based on international guidelines; (ii) implementation of a clinical and laboratory-based donor selection pipeline; and (iii) exploratory incorporation of metagenomic analysis of donor’s fecal microbiota.

### 2.2 Development of donor screening questionnaire and testing pipeline

Initially, a comprehensive literature review was performed to identify and compare current recommendations for FMT donor selection and biosafety. This review focused on three core components: clinical exclusion criteria, blood and serological testing, and microbiological screening of fecal samples. The screening tests developed by major national and international working groups were analyzed and compared, including those from the IAG HC-UFMG (Terra et al., 2020), the Netherlands Working Group (Terveer et al., 2020), the International Consensus Conference on FMT (Cammarota et al., 2019), the European FMT Working Group (Cammarota et al., 2017), and the Australian Consensus (Haifer et al., 2020). Based on this comparative analysis and additional literature, a customized screening questionnaire and testing pipeline were developed and implemented in the present work (**Supplementary Tables 1**, **2**).

### 2.3 Donor recruitment and clinical screening

Donor recruitment was conducted between July and September 2023 through public calls and institutional communication channels. Eligible volunteers were invited to complete a structured clinical screening questionnaire based on international FMT guidelines. Eligible donors were required to be between 18 and 50 years of age. Candidates presenting one or more exclusion criteria were not allowed to proceed to subsequent screening steps. Exclusion criteria included, but were not limited to, recent antibiotic use, chronic gastrointestinal diseases, metabolic or autoimmune disorders, recent infectious diseases, high-risk behaviors, and abnormal bowel habits. In addition, the clinical screening questionnaire included questions regarding illicit drug use, chronic medication use, occupational exposure to hazardous substances, and other potential risk factors associated with biological, toxicological or chemical exposure.

### 2.4 Laboratory tests

Candidates who met the clinical eligibility criteria were submitted to a comprehensive laboratory screening protocol, designed to assess general health status and exclude infectious agents potentially transmissible through fecal microbiota transplantation. Blood-based analyses included a complete blood count, serum electrolytes (sodium, potassium, calcium, magnesium), renal and hepatic function markers (urea, creatinine, AST, ALT, GGT, bilirubin), metabolic and nutritional parameters (glucose, vitamin B12, folic acid, vitamin D), and inflammatory markers (C-reactive protein). Serological screening comprised tests for HIV types I and II, hepatitis A (IgM/IgG), hepatitis B (HBsAg and anti-HBc IgG/IgM), hepatitis C (anti-HCV), human T-lymphotropic virus (HTLV I/II), cytomegalovirus (IgG/IgM), and Epstein–Barr virus (IgG/IgM).

Fecal samples were analyzed for enteric pathogens using conventional microbiological methods and molecular assays. These included coproculture and PCR-based detection of *Clostridioides difficile (C. difficile)* toxin A/B, rotavirus, adenovirus, SARS-CoV-2, herpes simplex virus I and II (IgG and IgM), and a multiplex PCR panel (FilmArray^®^) targeting bacterial, viral, and parasitic pathogens, such as *Campylobacter* spp., *Salmonella* spp., *Shigella*/EIEC, *Vibrio cholerae*, *Yersinia enterocolitica*, *C. difficile*, *Giardia lamblia*, *Entamoeba histolytica, Cryptosporidium, Cyclospora cayetanensis*, norovirus GI/GII, astrovirus, sapovirus, and adenovirus F40/41. Only candidates presenting normal results across all clinical, serological, biochemical, and microbiological tests were considered eligible donors for inclusion in the biobank.

### 2.5 Processing and storage of fecal material

Fecal material from eligible donors was processed under controlled laboratory conditions following standardized operating procedures. Prior to collection, eligible donors received a standardized collection kit containing sterile collection containers, disposable gloves, identification labels, and detailed written instructions describing the collection procedure and emphasizing aseptic handling practices. Donors were instructed to avoid contact between fecal material and external surfaces by using a single-use stool collection device (ColOff^®^) and to maintain samples refrigerated until transport to the laboratory. Samples were collected from donors’ homes shortly after evacuation and transported to the laboratory in insulated coolers containing ice packs. The total time elapsed between stool passage and laboratory processing did not exceed 6 hours. Upon arrival, samples were processed under atmospheric conditions in a laminar flow cabinet. Fecal material was homogenized in sterile phosphate-buffered saline (PBS), filtered through a sterile sieve to remove particulate debris, aliquoted into 10 mL fractions, and stored at −80 °C until use. All samples were labeled and documented to ensure traceability, and storage conditions were continuously monitored.

### 2.6 Shotgun metagenomics sequencing

Total genomic DNA from fecal samples obtained from eligible donors was extracted using the Quick-DNA™ HMW MagBead Kit (Zymo Research, Irvine, CA, USA), following the manufacturer’s instructions. DNA concentration was measured using the Qubit fluorometer (Thermo Fisher Scientific, Waltham, MA, USA), while DNA purity was assessed by NanoVue spectrophotometry (GE Healthcare, Chicago, IL, USA) based on the A260/A280 and A260/A230 ratios. DNA integrity was evaluated by agarose gel electrophoresis prior to library preparation.

Metagenomic sequencing libraries were prepared using the Rapid Barcoding Kit 24 v14 (SQK-RBK114.24; Oxford Nanopore Technologies, Oxford, UK), pooled, and sequenced for 48 h on a MinION device using an R10.4.1 flow cell. Raw reads were basecalled and demultiplexed using Guppy v4.3.0 (Oxford Nanopore Technologies). No extraction blank or negative sequencing control was included in the sequencing workflow.

### 2.7 Identification of antimicrobial resistance genes

Antimicrobial resistance determinants were identified directly from sequencing reads using the command-line version of the Resistance Gene Identifier (RGI, v6.0.3) against the Comprehensive Antibiotic Resistance Database (CARD). Read quality was evaluated using NanoPlot v1.40.0, followed by quality filtering using NanoFilt v2.8.0, retaining reads with a minimum mean quality score of Q10 and a minimum length of 500 bp. Only Perfect and Strict hits were retained for downstream analyses, whereas Loose hits were excluded to reduce the likelihood of false-positive assignments. Gene presence/absence, relative abundance, and cumulative abundance profiles were generated for descriptive comparisons across samples using R v4.3.2. Due to the exploratory nature of the study and the limited number of samples, no inferential statistical analyses were performed.

### 2.8 Genomic context and potential mobility of resistance determinants

To characterize the genomic context of ARGs and assess their potential mobility, sequencing reads were filtered at minimum mean quality score of Q8 and a minimum length of 1000 bp and were assembled using Flye v2.9.6. The resulting contigs were screened for ARGs using ABRicate v1.4.0 against the CARD database (≥90% identity and ≥80% coverage), and ARG-containing contigs were further characterized for plasmid association and mobility-related features using geNomad v1.12.0, MOB-suite v3.1.9, Bakta v1.9.4, and ISEScan v1.7.3. To assess sequence support for candidate contigs and ARGs, the reads were mapped to the corresponding Flye assemblies using Minimap2 v2.31, and read depth was calculated with SAMtools v1.23.1.

### 2.9 Data availability

The raw sequencing data generated in this study have been deposited in the NCBI Sequence Read Archive (SRA) under BioProject accession number PRJNA1499213.

### 2.10 Ethical considerations

The study was approved by the institutional review board under process number CAAE: 42603020.0.0000.0121. All participants provided written informed consent prior to sample collection and laboratory analyses.

## 3 Results

### 3.1 Donor recruitment and clinical eligibility

A total of 100 candidates were included in the clinical screening questionnaire during the recruitment period. Of these, 6 candidates met the clinical eligibility criteria and were referred to laboratory screening. Following biochemical, serological, and microbiological screening, two additional candidates were excluded, one due to the detection of toxigenic *C. difficile* by multiplex PCR (FilmArray^®^), while another candidate did not complete the laboratory screening process. Therefore, four participants were ultimately considered eligible for fecal microbiota donation and inclusion in the biobank. The donor selection workflow and the main exclusion criteria identified are illustrated in **Figure 1**. The demographic characteristics of the eligible donors are summarized in **Supplementary Table 3**.

**Figure 1:**
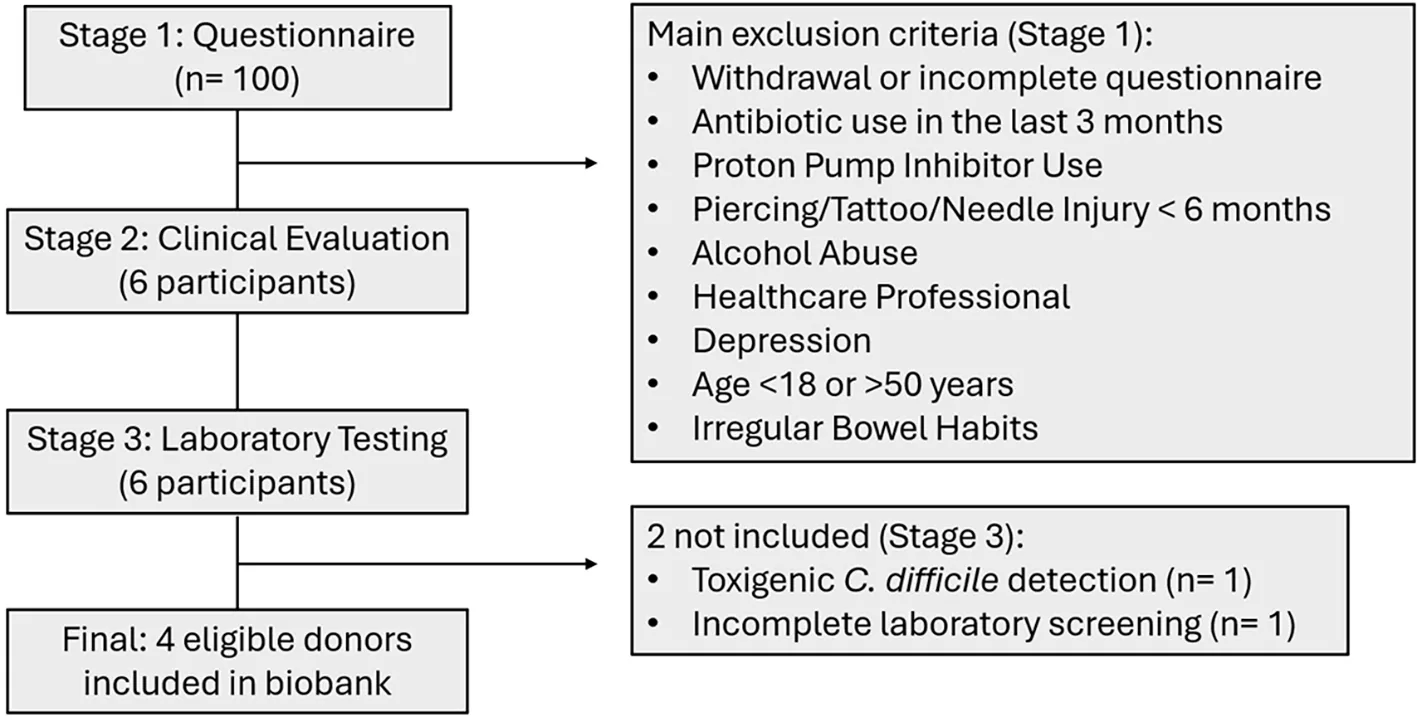
Flowchart illustrating the multistep donor selection process.

### 3.2 Resistome profile characterization

Sequencing generated 7.09 Gb of data across all samples (5.44 million reads; N50 = 2.55 kb), with read accuracy ranging from 79.6% to 84.7%, indicating overall good sequencing performance. Shotgun metagenomic analysis of fecal samples from eligible donors identified 180 different resistance-related gene entries in CARD dataset. Detection frequency varied between samples, with 137 genes identified in Sample 1, 87 in Sample 2, 103 in Sample 3, and 134 in Sample 4. Of these, only 58 were consistently detected in all samples, whereas 122 showed partial occurrence in different samples (**Figure 2A**). To improve biological interpretability, detected entries were grouped into two major categories: ARGs, comprising canonical resistance genes whose presence is directly associated with antimicrobial resistance phenotypes (n= 100), and variant-dependent loci, corresponding to conserved chromosomal targets in which resistance is typically mediated by specific sequence variants rather than gene presence alone (e.g., rRNA genes*, gyrA/parC/parE*, and *rpoB*-associated loci) (n=80). Among consensus genes, only eleven were classified as canonical ARGs (Core resistome), including *APH(3’)-IIIa, CfxA2, CfxA3, ErmF, mefA, AlaS* and five tetracycline resistance determinants *(tet (32*)*, tet (40*)*, tet(O), tet(Q), and tet(W)).*

**Figure 2:**
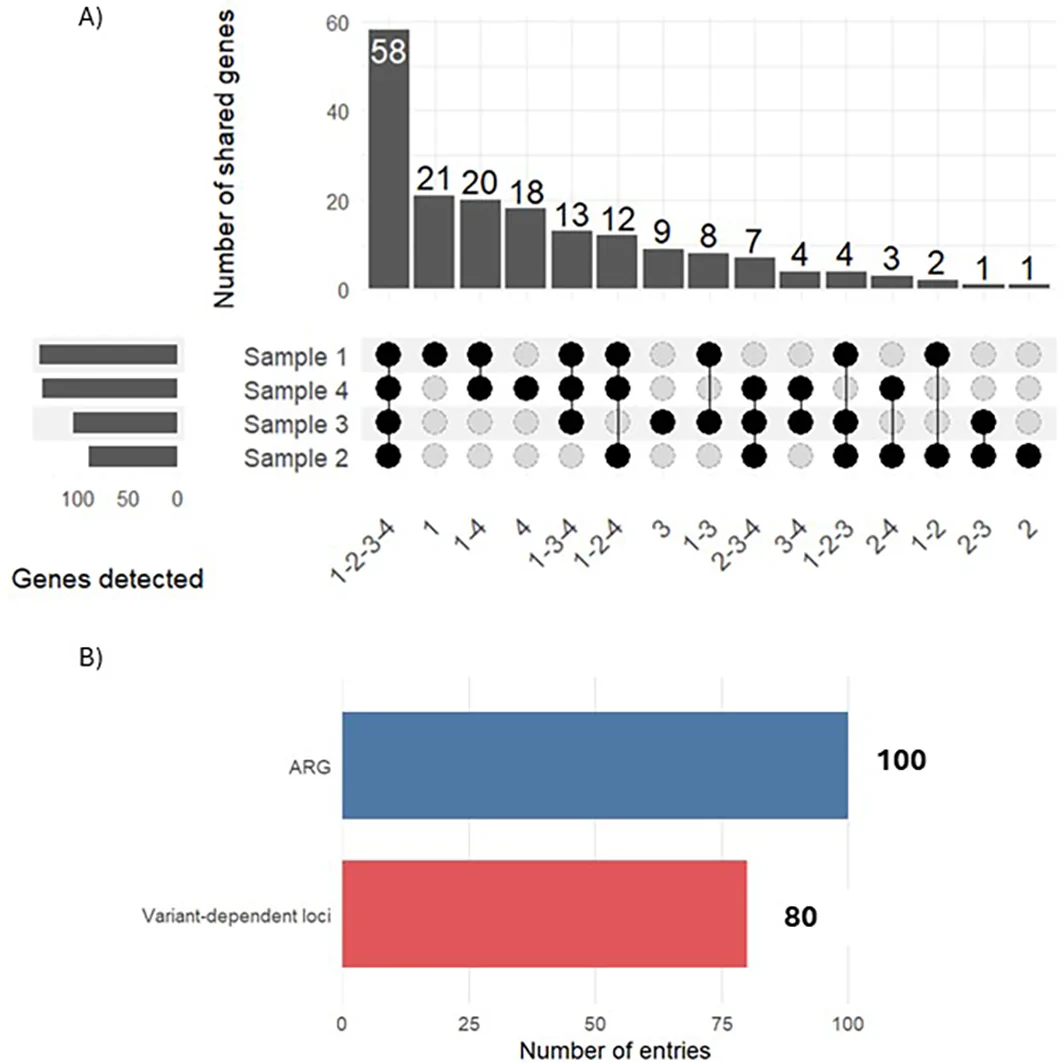
UpSet plot showing the intersection (number) of resistance-related genes detected across the four samples. The bar plot (top) indicates the number of genes shared among specific combinations of samples, while the dot matrix (bottom) represents the corresponding sample intersections. A total of 58 genes were detected in all four samples, while additional genes were shared among subsets of samples or were sample-specific **(A)**. Horizontal bars (left) indicate the total number of genes detected in each individual sample. Classification of resistance-related entries according to functional category **(B)**.

### 3.3 Taxonomic distribution and abundance of dominant antibiotic resistance genes

The taxonomic distribution of the most abundant ARGs across samples is shown in **Figure 3A**. A consistent set of dominant resistance genes was observed across all samples, with *tet(Q), tet(W)*, *tet(O), ErmF*, and *CfxA2* representing the most prominent determinants. These genes were strongly associated with specific bacterial taxa, indicating clear links between resistome composition and microbiota structure. In particular, *tet(Q)* was predominantly associated with *Bacteroides fragilis*, while *tet(W)* was mainly linked to *Bifidobacterium longum*. The gene *tet(O)* was primarily associated with *Campylobacter jejuni*, whereas *ErmF* was also largely attributed to *Bacteroides fragilis*. In contrast, *CfxA2* was associated with *Prevotella intermedia*, highlighting additional taxonomic contributors to β-lactam resistance.

**Figure 3:**
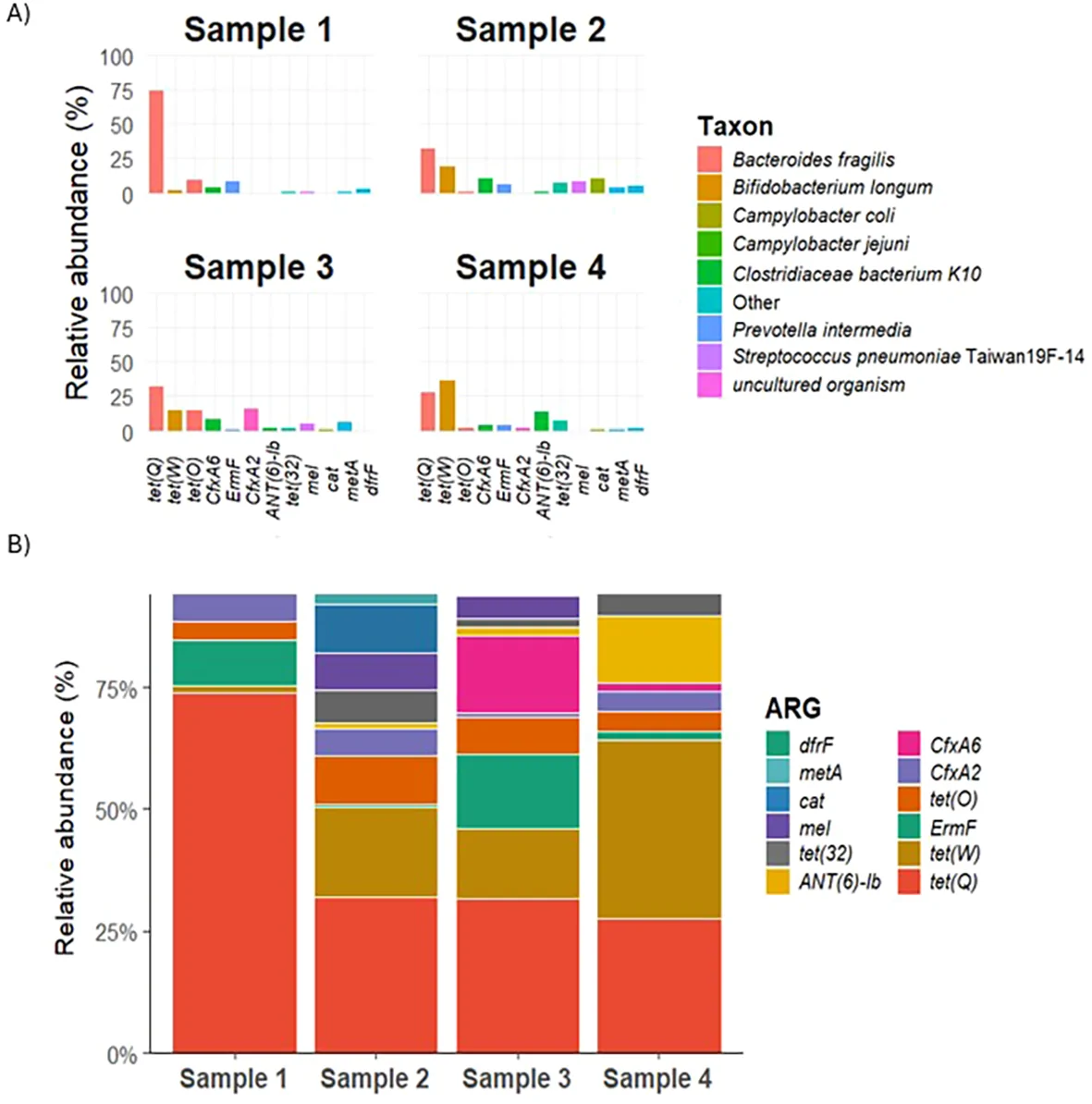
**(A)** Individual bar plots for each sample showing the distribution (%) of the 12 most abundant resistance genes, categorized by their associated bacterial taxa. The color legend indicates taxonomic group carrying the detected ARGs. **(B)** Stacked bar plot illustrating the distribution of 12 most abundant ARGs across all samples. Each color represents a specific ARG.

Although these genes were detected across all samples, their proportional abundance varied markedly (**Figure 3B**). Sample 1 was strongly dominated by *tet(Q)*, which accounted for 34.3% of ARG abundance, followed by *ErmF* (4.4%) and *APH(3’)-IIIa* (1.5%). In Sample 2, a more distributed profile was observed, with *tet(Q)* (9.1%), *tet(O)* (2.9%), *tet (32*) (1.9%), and *tet (40*) (1.7%) contributing to the resistome, along with macrolide resistance genes such as mel (2.2%). Sample 3 showed a more balanced composition, with *tet(Q)* (15.2%), *ErmF* (7.3%), *tet(W)* (7.0%), and *CfxA6* (7.6%) representing major contributors. In Sample 4, the dominance of *tet(Q)* was reduced (4.6%), with increased contributions from *tet(W)* (6.1%) and *ANT(6)-Ib* (2.3%), indicating a shift toward a more heterogeneous resistome structure.

### 3.4 Distribution of resistance classes and mechanisms

The resistome was predominantly composed of genes associated with aminoglycoside resistance (32.9%) and tetracycline resistance (28.9%), which together accounted for more than half of the total ARG abundance. Genes related to multidrug resistance also represented a substantial fraction (19.3%), indicating the presence of broad-spectrum resistance determinants. Other classes were detected at lower frequencies, including cephalosporin (5.1%), peptide (3.4%), and elfamycin (2.3%), while macrolide (1.2%) and streptogramin (1.1%) resistance genes contributed minimally to the overall resistome.

The functional mechanisms underlying resistance are summarized in **Figure 4B**. The resistome was strongly dominated by target alteration mechanisms (58.2%), followed by target protection (26.8%), indicating that resistance in these samples is primarily mediated through modifications of antibiotic targets rather than drug degradation or efflux. Enzymatic inactivation mechanisms accounted for 8.4%, while efflux systems contributed 2.9% of the total ARG abundance. Additional mechanisms, including target replacement (1.2%) and unclassified categories (2.4% unknown; 0.1% other), were detected at low frequencies.

**Figure 4:**
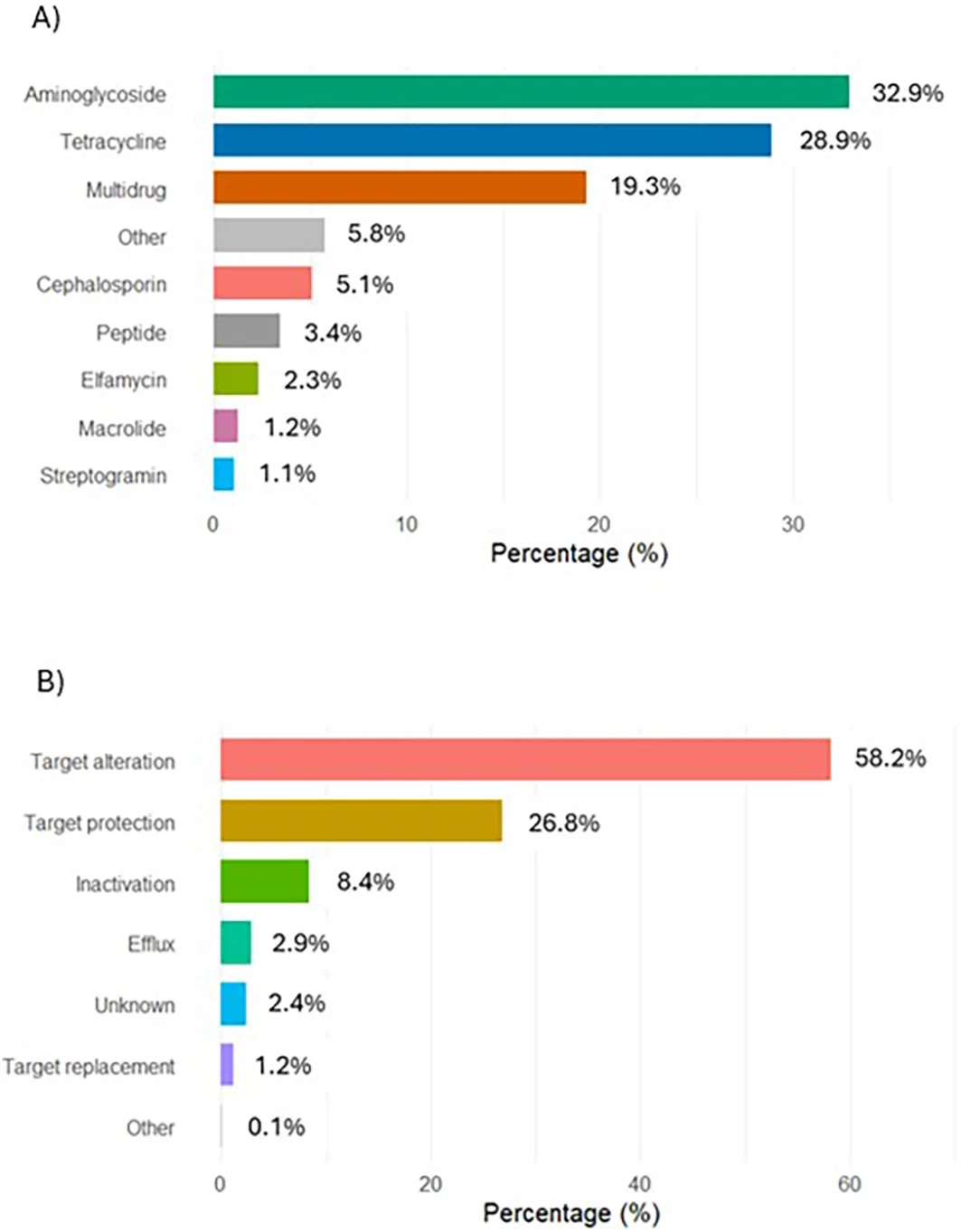
Distribution of resistance genes according to the class of antibiotics **(A)**. Relative abundance of antimicrobial resistance mechanisms identified across the samples **(B)**.

### 3.5 Genomic context supports potential mobility of ARGs

Because the presence of an ARG alone does not indicate its potential for transmission, we further examined the genomic context of resistance determinants. Four ARG-containing contigs showed features compatible with potential mobility, including putative plasmid associations and mobility related features in chromosomal contexts (**Supplementary Table 4**).

In Sample 1, a *tet(Q)* gene was located on a circular contig that was classified as a plasmid by MOB-Recon and geNomad (score: 0.9949). The contig contained a gene annotated as *repA* and a gene encoding an IS66 family transposase, with the transposase located approximately 1.1 kb downstream of the *tet(Q)* coding region. In Sample 4, an *APH(3’)-IIIa* gene was also located on a circular contig classified as plasmid (score 0.9927) and contained genes encoding plasmid recombination proteins and a MOBV relaxase. In the same sample, a *tet(Q)* gene was located approximately 5.4 kb upstream of a region containing multiple genes associated with conjugative transfer (*traO, traN, traM, traJ, traG, and traD*) and a relaxase. In Sample 3, *tet(Q)* and *ermF* genes were located on the same chromosomal contig, with an IS30 family transposase gene positioned immediately upstream of *ermF* and approximately 1.1 kb downstream of *tet(Q)*. These findings indicate that a subset of resistance determinants identified in donor feces occurred within genomic contexts compatible with potential dissemination.

## 4 Discussion

A central challenge in FMT implementation worldwide remains the identification of suitable donors. Our findings confirm that donor selection is a highly restrictive process, with only a minority of screened candidates ultimately qualifying for donation. Similar donor approval rates have been reported in international biobanking initiatives, reflecting the stringency required to minimize transmissible risks (Cammarota et al., 2017; Terra et al., 2020). This reinforces the importance of structured, transparent, and reproducible selection workflows as a foundational element of any FMT program. By integrating clinical questionnaires, biochemical and serological testing, and microbiological screening, our workflow aligns with major international guidelines while being operationally feasible within public and private healthcare infrastructures in Brazil (Cammarota et al., 2019). This aspect is particularly relevant in low- and middle-income countries, where the implementation of microbiota-based therapies often faces infrastructural and regulatory barriers.

An important contribution of this study is the incorporation of metagenomic sequencing and ARG profiling as an additional source of information for potential donor–recipient selection and risk assessment. In this work, we identified antimicrobial resistance determinants involving clinically relevant antibiotic classes, such as tetracyclines and aminoglycosides, within major members of the gut microbiota, with some occurring in genomic contexts compatible with potential mobilization, even among donors who had undergone rigorous screening.

As expected, tetracycline resistance determinants such as *tet(Q)* and *tet(W)* were highly prevalent and primarily associated with *Bacteroides fragilis* and *Bifidobacterium longum*, respectively, reinforcing the well-established role of these taxa as major reservoirs of tetracycline resistance in the human gut (Shoemaker et al., 2001; Hu et al., 2013). The relatively high abundance of *ermF*, a gene associated with cross-resistance to macrolides, lincosamides, and streptogramin B antibiotics (MLS_b_ phenotype), and linked to *Bacteroides fragilis*, further highlights the role of this species as a reservoir of resistance determinants (Shoemaker et al., 2001). Notably, *tet(Q)* and *ermF* occurred together in a chromosomal context containing an IS30 family transposase in Sample 3, sharing features with resistance architectures described in Bacteroides conjugative transposons, including the CTnDOT family, which carries both determinants and contains regions involved in mobilization and conjugative transfer (Shoemaker et al., 2001; Jeters et al., 2009). Other *tet(Q)* and ARG containing contigs showed complementary features compatible with potential mobility, including proximity to conjugative transfer machinery and association with circular plasmid contigs containing plasmid replication, recombination, or relaxase features. Together, these findings indicate that some resistance determinants occurred in genomic environments compatible with potential mobilization; however, assessment of active horizontal gene transfer was beyond the scope of this study.

Although the mobility and horizontal transfer of ARGs are important concerns, the potential for bacteria carrying ARGs in donor feces to persist, translocate, and cause infection in immunocompromised recipients should not be overlooked. This concern is illustrated by the seminal work of DeFilipp et al., which traced the transmission of an extended-spectrum β-lactamase (ESBL)-producing *Escherichia coli* from donor material to a recipient who subsequently developed bacteremia and died (DeFilipp et al., 2019). In contrast, Woodworth et al. showed that FMT can also promote sustained decolonization of multidrug-resistant organisms, with replacement of baseline ESBL-producing bacteria by susceptible strains (Woodworth et al., 2023). These contrasting findings suggest that the clinical implications of ARGs in FMT depend on the broader donor–recipient context, including the bacterial host and genomic context, but particularly the recipient’s clinical condition and anticipated antimicrobial exposure.

In this context, ARG profiling could be used as a complementary layer of donor–recipient assessment rather than as an exclusion criterion, helping to identify cases that may warrant further investigation through phenotypic antimicrobial susceptibility testing or deeper genomic characterization. For example, when clinically relevant resistance determinants are identified, decision makers could consider further investigation of donor feces based on existing guidance for surveillance of carbapenem-resistant Gram-negative bacteria in health care settings (World Health Organization, 2017) This approach could potentially be extended to ESBL producing organisms until further studies specifically evaluating antimicrobial resistance related risks in FMT become available. Future longitudinal studies integrating donor–recipient metagenomic profiling, strain tracking, and clinical outcomes will be important to determine how donor resistance profiles influence the clinical effects of FMT and to refine this risk assessment approach.

Beyond donor screening, alternative FMT formulations may provide complementary strategies to improve biosafety. Sterile fecal filtrates and defined microbial consortia, for example, do not rely on the transfer of viable bacterial communities and may therefore reduce some of the risks associated with the introduction of donor-derived organisms (Ott et al., 2017; Feuerstadt et al., 2022). Early clinical evidence suggests that some of these approaches may retain therapeutic efficacy, although their safety and effectiveness require validation in larger and more diverse clinical settings (Ott et al., 2017; Kao et al., 2025). Together, approaches that combine improved biosafety with greater standardization may facilitate the broader and safer implementation of microbiota-based therapies.

## 5 Limitations and perspectives

Some limitations of this study should be acknowledged. First, the number of participants who completed the full donor selection pipeline and the number of donors subjected to metagenomic sequencing were relatively small, limiting the assessment of individual variability and the generalizability of ARG profiles to the broader regional population. Each sequenced donor represented a single independent biological sample; therefore, differences in ARG occurrence and proportional abundance across samples should be interpreted as descriptive and exploratory. In addition, the donor screening workflow did not include laboratory based toxicological analyses for environmental contaminants, pharmaceutical residues, pesticides, heavy metals, or other chemical exposures, although medication use and potential exposure risks were assessed through the clinical screening questionnaire.

Second, the detection of ARGs was based on sequence matching and was not complemented by phenotypic antimicrobial susceptibility testing or functional characterization. Although genomic context analysis identified ARG-containing contigs with features compatible with potential mobility, these observations are insufficient to demonstrate active mobilization or horizontal gene transfer, which were not directly assessed in this study. In addition, the limited number of sequenced donors and the use of assembled metagenomic contigs limit the ability to generalize these findings or fully resolve the genomic organization and strain-level distribution of the identified resistance determinants.

Finally, the use of a single sequencing platform and analytical pipeline represents a methodological limitation. The sequencing accuracy obtained with the Oxford Nanopore platform may influence sequence alignment and ARG assignment and should be considered when interpreting the results. Future studies incorporating larger donor cohorts, complementary sequencing approaches, characterization of the genomic context and mobility of ARGs, and longitudinal donor–recipient analyses integrating microbiome profiles with clinical outcomes will be important to better define the clinical relevance of ARG profiling in microbiota-based therapies.

## Data Availability

The data presented in this study have been deposited in the National Center for Biotechnology Information (NCBI) repository under accession number PRJNA1499213.
