# Supplementary material for: Identification of antibiotic resistance genes in fecal microbiota selected donors during the establishment of a biobank in the south of Brazil
Source: Front Antibiot. 2026 Sep 25;5:1905540. doi: 10.3389/frabi.2026.1905540 (PMC13649761; doi:10.3389/frabi.2026.1905540)
Supplement: Supplementary file 1 [file DataSheet1.pdf]

Supplementary Data

Supplementary Table S1. Comparison of exclusion criteria for fecal microbiota transplantation donor screening across international guidelines

Exclusion Criteria	UFMG (2020)	Amsterdam (2013)	International (2019)	European (2016)	Australian (2020)
Known uncontrolled systemic infection at the time of donation	X		X	X	
Use of illicit drugs	X	X	X	X	X
High-risk sexual behavior (anonymous partners; contact with sex workers, drug users, or individuals with HIV, viral hepatitis, or syphilis; sex work; history of sexually transmitted infections)	X	X	X	X	X
Previous receipt of organ/tissue transplant	X	X	X	X	
Previous receipt of blood products	<6 months	X		<12 months	
Recent needlestick injury	<6 months	X		<6 months	<3 months
Recent tattoo, piercing, earrings, acupuncture	<6 months	X	<6 months	<6 months	<3 months
Recent parasitic infection or gastrointestinal infection (e.g., rotavirus, *Giardia lamblia*)			≤2 months	X	
Recent travel (<6 months) to tropical countries or regions with high risk of infectious diseases or traveler's diarrhea		X	X	X	<3 months
Recent vaccination with live attenuated virus (if potential transmission risk exists)	<2 months		≤2 months	<6 months	
Healthcare workers (risk of multidrug-resistant organism transmission)	X	X		X	
Occupational exposure to animals (risk of zoonotic infections)	X			X	
History of IBS, IBD, chronic functional constipation, celiac disease, or other chronic gastrointestinal disorders	X	X	X	X	X
History of chronic systemic autoimmune diseases with gastrointestinal involvement	X		X	X	X
History or high risk of gastrointestinal cancer or polyposis	X	X	X	X	X
Recent onset of diarrhea or hematochezia	X		X	X	
History of neurological/neurodegenerative diseases	X		X	X	
History of psychiatric disorders	X		X	X	
Overweight/obesity	BMI ≥25		BMI >30	BMI >25	BMI >30

Exclusion Criteria	UFMG (2020)	Amsterdam (2013)	International (2019)	European (2016)	Australian (2020)
Recent exposure to antibiotics, immunosuppressants, or chemotherapy	<3 months	Antibiotics	X	<3 months	<3 months
Chronic proton pump inhibitor use	X		X	X	
Diarrhea (loose/liquid stools >3 times/day)	<6 months			X	<1 month
Recent illness or systemic symptoms (fever, sore throat, lymphadenopathy)	<2 weeks	Fever <2 weeks		X	
Age limits	<18 or >50	>60	>60	>60	<16 or >60
History of hepatitis A			X		6 months
Infection with multidrug-resistant bacteria					<3 months
Gastrointestinal surgery	X				X
Metabolic syndrome, diabetes	X	X			X
Known history or risk factors for infectious diseases		X	X		
Recent hospitalization or stay in long-term care facilities	>2 days in last 3 months		X		
Recent needlestick injury	<6 months	X	≤6 months		
History of receiving growth hormone, bovine insulin, or coagulation factor concentrates		Growth hormone	X		
Known exposure or history of HIV-1/2, hepatitis B/C, syphilis, HTLV-1/2, malaria, Chagas disease, cutaneous tuberculosis, or mucosal herpes	X	X			
Risk factors for Creutzfeldt–Jakob disease (personal/family history, corneal transplant, cadaveric pituitary hormone use, bovine insulin use, nosocomial exposure, residence in UK/Ireland >3 months between 1980–1996 or >5 years in Europe since 1980)	X	X			
Sexual contact with individuals with HIV or viral hepatitis; men who have sex with men; partners of bisexual men	X	X			
New sexual partner within the last 12 months	X	X			
Moderate to severe malnutrition	X				

"X" indicates inclusion of the criterion; time restrictions are specified where applicable. Abbreviations: AST, aspartate aminotransferase; ALT, alanine aminotransferase; GGT, gamma-glutamyl transferase; CRP, C-reactive protein; HIV, human immunodeficiency virus; HTLV, human T-lymphotropic virus; CMV, cytomegalovirus; EBV, Epstein–Barr virus; PCR, polymerase chain reaction.
