# Supplementary material for: Identification of antibiotic resistance genes in fecal microbiota selected donors during the establishment of a biobank in the south of Brazil
Source: Front Antibiot. 2026 Sep 25;5:1905540. doi: 10.3389/frabi.2026.1905540 (PMC13649761; doi:10.3389/frabi.2026.1905540)
Supplement: Supplementary file 2 [file DataSheet2.pdf]

Supplementary Table S2. Laboratory screening workflow for donor eligibility

Screening Type	Test Category	Specific Tests / Targets
**General Health (Blood Tests)**	Hematology	Complete blood count
	Electrolytes	Sodium, potassium, calcium, magnesium
	Renal function	Urea, creatinine
	Liver function	AST, ALT, GGT, total and fractionated bilirubin
	Metabolic/Nutritional markers	Glucose, vitamin B12, folic acid, vitamin D
	Inflammatory marker	C-reactive protein (CRP)
**Serological Screening (Blood-borne pathogens)**	Viral serology	HIV-1/2, Hepatitis A (IgM/IgG), Hepatitis B (HBsAg, anti-HBc IgG/IgM), Hepatitis C (anti-HCV), HTLV-1/2
	Herpesviruses	Cytomegalovirus (CMV IgG/IgM), Epstein–Barr virus (EBV IgG/IgM), Herpes simplex virus I/II (IgG/IgM)
**Stool Microbiological Screening**	Conventional microbiology	Coproculture
	Toxin detection	*C. difficile* toxin A/B
**Molecular Screening (PCR-based assays)**	Targeted PCR	SARS-CoV-2, rotavirus, adenovirus
	Multiplex PCR panel (FilmArray®)	Bacteria: *Campylobacter spp.*, *Salmonella spp.*, *Shigella/EIEC*, *Vibrio cholerae*, *Yersinia enterocolitica*, *C. difficile*<br>Parasites: *Giardia lamblia*, *Entamoeba histolytica*, *Cryptosporidium*, *Cyclospora cayetanensis*<br>Viruses: Norovirus GI/GII, astrovirus, sapovirus, adenovirus F40/41

Abbreviations: AST, aspartate aminotransferase; ALT, alanine aminotransferase; GGT, gamma-glutamyl transferase; CRP, C-reactive protein; HIV, human immunodeficiency virus; HTLV, human T-lymphotropic virus; CMV, cytomegalovirus; EBV, Epstein–Barr virus; PCR, polymerase chain reaction.
