# Supplementary material for: Identification of antibiotic resistance genes in fecal microbiota selected donors during the establishment of a biobank in the south of Brazil
Source: Front Antibiot. 2026 Sep 25;5:1905540. doi: 10.3389/frabi.2026.1905540 (PMC13649761; doi:10.3389/frabi.2026.1905540)
Supplement: Supplementary file 3 [file DataSheet3.pdf]

Supplementary Table S3. Demographic and general characteristics of eligible fecal microbiota donors

Characteristics	Donor 1	Donor 2	Donor 3	Donor 4
**Age, years**	21	30	35	23
**Sex**	Female	Male	Male	Female
**Place of residence**	Florianópolis, Brazil	Florianópolis, Brazil	Florianópolis, Brazil	Florianópolis, Brazil
**History of blood donation**	Yes (2023)	No	Yes (2022)	No

Note: Years in parentheses indicate the most recent blood donation.
