# Supplementary material for: Identification of antibiotic resistance genes in fecal microbiota selected donors during the establishment of a biobank in the south of Brazil
Source: Front Antibiot. 2026 Sep 25;5:1905540. doi: 10.3389/frabi.2026.1905540 (PMC13649761; doi:10.3389/frabi.2026.1905540)
Supplement: Supplementary file 4 [file DataSheet4.pdf]

Supplementary Table S4. Evidence supporting potential mobility of ARGs

Sample	Contig	Length (bp)	Flye cov	ARG identity / coverage	Plasmid evidence	Mobility features	Mobility identity / coverage	Read depth
1	2852	8.311	370×; circular	*tet(Q):* 96,35% / 100%	Circular; MOB-Recon: plasmid; geNomad score 0.9947	*repA, mobB*/MOBB; IS66-family transposase	IS66: 88,0% / 100%; *repA*: 99,0% / 89,0%; *mobB: 90,7% / 93,1%*	~377× contig; ~426× ARG
3	522	22.310	8×; linear	*tet(Q):* 97,01% / 99,75%; *ErmF:* 98,76% / 99,88%	Not detected; MOB-Recon: chromosomal	IS30-related region	IS30: 100% / 83,6%	~63,7×
4	170	70.569	18×; linear	*tet(Q):* 97,47% / 100%	Not detected; MOB-Recon: chromosomal	Conjugative transposon machinery + relaxase/mobilisation nuclease	*TraO* 92,1% / 86,3%; *TraN* 96,3% / 98,2%; *TraM* 94,4% / 91,8%; *TraJ* 98,9% / 82,4%; *TraG* 97,5% / 81,1%; *TraD* 96,8% / 97,3%; relaxase 92,3% / 91,6%	~22,1× contig; ~20,8× ARG
4	2066	6.006	11×; circular	*APH(3')-IIIa:* 99,62% / 99,75%	Circular; MOB-Recon: plasmid; geNomad score 0.9929	Plasmid recombination proteins	Plasmid recombination protein; MOBV relaxase 100% / 91,4%	~12,0× contig; ~12,0× ARG

Note: ARG identity/coverage refers to nucleotide sequence identity and alignment coverage. Flye cov, assembly coverage estimated by Flye; read depth, sequencing read depth calculated by read mapping to the corresponding contig. Plasmid classification was based on MOB-Recon and geNomad. Mobility features were identified through sequence annotation and homology-based analyses. Abbreviations: bp, base pairs; ARGs, antibiotic resistance genes; IS, insertion sequence; MOB, mobilization; Tra, conjugative transfer protein.
